# Regional expression of *HOXA4 *along the aorta and its potential role in human abdominal aortic aneurysms

**DOI:** 10.1186/1472-6793-11-9

**Published:** 2011-05-31

**Authors:** John H Lillvis, Robert Erdman, Charles M Schworer, Alicia Golden, Kimberly Derr, Zoran Gatalica, Laura A Cox, Jianbin Shen, Richard S Vander Heide, Guy M Lenk, Leigh Hlavaty, Li Li, James R Elmore, David P Franklin, John L Gray, Robert P Garvin, David J Carey, Wayne D Lancaster, Gerard Tromp, Helena Kuivaniemi

**Affiliations:** 1 Center for Molecular Medicine and Genetics, Wayne State University School of Medicine, Detroit, Michigan, USA; 2 Sigfried and Janet Weis Center for Research, Geisinger Clinic, Danville, 100 North Academy Avenue, Pennsylvania 17822-2610, USA; 3 Department of Pathology, Creighton University School of Medicine, Omaha, Nebraska, USA; 4 Southwest National Primate Research Center, San Antonio, Texas, USA; 5 Department of Genetics, Texas Biomedical Research Institute, San Antonio, Texas, USA; 6 Department of Internal Medicine, Wayne State University School of Medicine, Detroit, Michigan, USA; 7 Department of Pathology, Louisiana Health Sciences Center, New Orleans, Louisiana, USA; 8 Office of Wayne County Medical Examiner, Detroit, Michigan, USA; 9 Department of Vascular and Endovascular Surgery, Geisinger Clinic, Danville, Pennsylvania, USA; 10 Department of Obstetrics and Gynecology, Wayne State University School of Medicine, Detroit, Michigan, USA

## Abstract

**Background:**

The infrarenal abdominal aorta exhibits increased disease susceptibility relative to other aortic regions. Allograft studies exchanging thoracic and abdominal segments showed that regional susceptibility is maintained regardless of location, suggesting substantial roles for embryological origin, tissue composition and site-specific gene expression.

**Results:**

We analyzed gene expression with microarrays in baboon aortas, and found that members of the HOX gene family exhibited spatial expression differences. *HOXA4 *was chosen for further study, since it had decreased expression in the abdominal compared to the thoracic aorta. Western blot analysis from 24 human aortas demonstrated significantly higher HOXA4 protein levels in thoracic compared to abdominal tissues (*P *< 0.001). Immunohistochemical staining for HOXA4 showed nuclear and perinuclear staining in endothelial and smooth muscle cells in aorta. The *HOXA4 *transcript levels were significantly decreased in human abdominal aortic aneurysms (AAAs) compared to age-matched non-aneurysmal controls (*P *< 0.00004). Cultured human aortic endothelial and smooth muscle cells stimulated with INF-γ (an important inflammatory cytokine in AAA pathogenesis) showed decreased levels of HOXA4 protein (*P *< 0.0007).

**Conclusions:**

Our results demonstrated spatial variation in expression of HOXA4 in human aortas that persisted into adulthood and that downregulation of *HOXA4 *expression was associated with AAAs, an important aortic disease of the ageing population.

## Background

Many vascular diseases have the tendency to manifest at specific sites in the vasculature. Aortic aneurysms are one such disease with approximately 90% developing between the renal arteries and iliac bifurcation. Aneurysms develop less frequently in the ascending and descending thoracic aorta, and are distinct from abdominal aortic aneurysms (AAAs) in prevalence, risk factors, genetics and histology [1].

Several intrinsic structural differences between the abdominal and thoracic aorta may contribute to aneurysm susceptibility [2,3]. The abdominal aorta has a narrower diameter and contains several major branch points, both of which result in turbulent blood flow and decreased shear stress. Areas of low shear stress are more susceptible to atherosclerotic lesions. In addition, the abdominal aortic wall is thinner than that of the thoracic aorta, contains fewer elastic lamellae, as well as decreased amounts of the structural proteins, elastin and collagen. These structural differences may confer greater susceptibility to injury and dilatation to the abdominal aorta.

There are also important embryological differences between the thoracic and abdominal aorta. Neural crest-derived smooth muscle cells (SMCs) populate the ascending thoracic aorta, while mesoderm-derived cells are found in the abdominal aorta [4,5]. These distinct cell populations respond differently to growth factors such as TGF-β [4,5].

One group of genes that may contribute to regional heterogeneity in the vasculature is the homeobox (HOX) transcription factors, which are known to control embryonic spatial patterning. In multiple adult cell types microarray analyses found a correlation between HOX gene expression and anatomic site of origin [6,7]. Animal experiments have demonstrated distinct HOX gene expression domains within the vasculature [8]. Finally, HOX genes regulate proliferation and differentiation of vascular cells [9].

Characterization of regional heterogeneity in the aorta may help identify novel mechanisms of AAA formation. In the current study we used microarray-based expression profiling to identify genes whose expression differed along the aorta in baboons. We identified several members of the HOX family of transcription factors with spatial expression changes, as well as reduced mRNA levels in samples from human AAA. Further studies on *HOXA4 *with human aortic samples confirmed its spatial expression pattern and identified it as a novel gene of interest for human AAA pathogenesis.

## Methods

### Baboon samples

**Figure 1:**
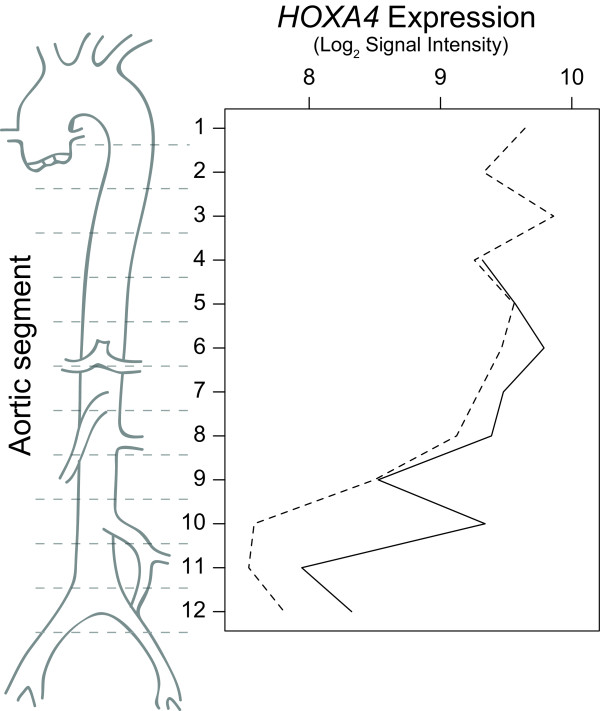
***HOXA4 *mRNA expression in baboon aorta**. *HOXA4 *levels were measured using microarrays in 12 different segments taken along the length of two baboon aortas.

Full thickness aortic wall tissue specimens were collected from baboons (*Papio hamadryas anubis*) (N = 3) euthanized for old age at the Southwest National Primate Research Center, San Antonio, Texas, USA. The aortas were divided into 10-12 segments (Figure 1), placed in RNAlater (Ambion, Austin, TX, USA) solution and processed for RNA isolation. Aortic segments were assigned similar numbers based on anatomical landmarks including the aortic arch (section 1), renal arteries (section 8) and iliac bifurcation (section 12).

### Human samples

Full thickness aortic wall tissue specimens (Additional file 1, Table S1 and Additional file 2, Table S2) were collected from patients undergoing AAA repair (N = 18) operations at the Harper University Hospital, Detroit, Michigan or at Geisinger Medical Center, Danville, Pennsylvania, USA, and from controls without aneurysms (N = 43) at autopsies or through the National Disease Research Interchange (NDRI, Philadelphia, PA, USA; http://www.ndriresource.org). Samples were stored in RNAlater for RNA isolations (Ambion), fixed in phosphate-buffered formalin (and embedded in paraffin) for histological and immunohistochemical analyses, snap-frozen in liquid nitrogen for protein analyses, or embedded in O.C.T.-media (Sakura-Finetek USA, Inc., Torrance, CA, USA) for immunohistochemical analyses. The collection of the human tissues was approved by the Institutional Review Boards of Wayne State University, Detroit, Michigan, USA, and Geisinger Clinic, Danville, Pennsylvania, USA.

### RNA isolation, microarray studies, and RT-PCR

We isolated total RNA from baboon and human blood vessels as described previously [10]. Quality of RNA was assessed by Bioanalyzer (Agilent Technologies, Inc., Santa Clara, CA, USA) and quantified using a NanoDrop instrument (Nanodrop products, Wilmington, DE, USA).

Our laboratory used two microarray platforms to generate global mRNA expression profiles for both aneurysmal and non-aneurysmal human abdominal aorta. The details on these studies have been described previously [11], and the microarray data can be obtained at the Gene Expression Omnibus (GEO) database (Series# GSE7084; http://www.ncbi.nlm.nih.gov/geo/). Details on the samples used for the microarray studies can be found in Additional file 2, Table S2. The AAA case (N = 6) and control (N = 7) groups were matched for age, sex and ethnicity (AAA mean age = 67.8 +/- 7.3 y; control mean age = 65.6 +/- 12.1 y; *P *= 0.69, Additional file 2, Table S2).

For the baboon experiments 500 ng of total RNA was reverse transcribed and 1.5 μg of cRNA was hybridized to either Sentrix Human WG-6 version 1 (N = 1) or HumanRef-8 version 2 microarrays (N = 2) (Illumina). QC procedures included examination of raw and adjusted intensity histograms and principal component analysis for systematic bias. We used the "Detection Score" to determine expression in the Illumina platform. The Illumina data were adjusted ("normalized") using a cubic spline function. To identify genes with relative increases or decreases in expression along the length of the aorta, linear regression was performed using the R statistical package [12,13]. 99% confidence intervals (CI) were constructed for the calculated slope and those probes with a CI not crossing zero were considered to have increasing or decreasing expression.

Real-time quantitative RT-PCR for *HOXA4 *on RNA isolated from human AAA samples (N = 12) and control (N = 12) abdominal aorta samples (Additional file 2, Table S2) was performed using a commercially available assay (catalog number HS01573270_m1) according to the manufacturer's instructions (Applied Biosystems, Life Technologies Corporation, Carlsbad, CA, USA). AAA case and aortic control groups did not differ significantly by age (AAA mean = 69.9 +/- 10.2 y; control mean = 65.6 +/-12.1 y; *P *= 0.27; Additional file 2, Table S2). Levels of 18S RNA were also determined to standardize the results. All experiments were run in triplicate. After PCR, amplification plots were inspected and baselines and threshold values were set for signals using Sequence Detection System software according to the manufacturer's recommendations (Applied Biosystems). The threshold cycle numbers (C_T_) were computed for each well. For calculating the ΔC_T _values for each well and processing the results further, the raw data were transferred into a Microsoft Access database [14]. Briefly, 18S amplification C_T _values were subtracted from the *HOXA4 *C_T _values for each sample to calculate the ΔC_T _[14]. The *P*-values were calculated using the Wilcoxon rank-sum test. The results of the statistical analysis were summarized using the Program R [13] and are presented as a box-and-whiskers plot.

### Western blot analysis of aortic proteins

Between 200 and 400 mg of flash-frozen aortic tissue was homogenized in buffer consisting of 50 mM Tris, 100 mM NaCl, 5 mM EDTA, 5 mM EGTA, 1% Triton X-100, 0.1% SDS, and 1x Mammalian Protease Inhibitor Cocktail (Catalog Number P8340, Sigma-Aldrich, St. Louis, MO, USA), pH 7.4. Additionally, either 1x HALT Phosphatase Inhibitor (Fisher Scientific, Pittsburgh, PA, USA) or Phosphatase Inhibitor Cocktail II (Sigma-Aldrich) was included in the homogenization buffer. Homogenized samples were centrifuged and supernatants were used for analysis. Protein concentrations were determined using the bicinchoninic bicinchoninic acid (BCA) assay from Pierce following the manufacturer's instructions (Thermo Fisher Scientific, Inc., Rockford, IL, USA). A total of 20 μg of protein was used for HOXA4 and ACTB, and 0.5 μg for ACTA2 analyses. Samples were loaded onto precast 4-15% gradient polyacrylamide gels (Bio-Rad Laboratories, Hercules, CA) and electrophoresed for 35 minutes at 200 V. Proteins were transferred to nitrocellulose membranes in a buffer containing 20% methanol, 0.19 M glycine, and 25 mM Tris in a Bio-Rad Mini Trans-Blot Cell at 100 V for 1 hour. After protein transfer, blots were fixed at room temperature for 15 minutes in a solution containing 25% isopropanol and 10% acetic acid, rinsed briefly with 0.1 M Tris, 0.154 M NaCl, pH 7.5 with 0.1% Tween 20 (TBS-Tween or TBST) and blocked for 45 minutes at room temperature in 5% skim milk in TBST. Blots were then transferred to heat-sealable bags and incubated overnight at 4°C with primary antibody (HOXA4: ab26097 from Abcam, Cambridge, MA; ACTAB: A1978 from Sigma-Aldrich, St. Louis, MO; and ACTA2: ab5694 from Abcam) diluted in blocking solution. Following washes the blots were incubated with HRP-conjugated IgG preabsorbed species-specific secondary antibody (Santa Cruz Biotechnology, Inc., Santa Cruz, CA, product number sc-2054 and GE Healthcare Biosciences, Piscataway, NJ, product number NXA931) diluted to 1:25,000 in TBST containing 4% serum of the secondary antibody species. After a final washing in TBST, blots were visualized using Pierce SuperSignal West Pico enhanced chemiluminescent substrate according to manufacturer's instructions and a Bio-Rad ChemiDoc Imaging system. Autoradiography film exposures were also obtained for each blot (Additional file 3, Figure S1).

The HOXA4 antibody (ab26097, Abcam) was produced against a synthetic peptide derived from residues 1 - 100 of human HOXA4. The immunogen is specific for HOXA4 in NCBI Blast search and shows no similarity to other HOX proteins. The same antibody has been used previously by Ota et al. [15].

Blots were quantified using Quantity One^® ^software (Bio-Rad). An intensity-per-area value was obtained for each sample band as well as for a control sample loaded on all blots. Band intensities were adjusted between blots using control sample intensities. Intensity values adjusted for expression of either ACTA or ACTB were input into the R statistical package [13]. A Wilcoxon signed rank test was used to compare the intensity values of protein bands from thoracic and abdominal aortic wall samples. Linear regression with stepwise backwards elimination was used to evaluate age, race and sex as predictors of ATCB-adjusted HOXA4 protein levels.

### Immunohistochemical staining of aortic tissues

Immunohistochemical staining of O.C.T-embedded frozen tissues was carried out using the VECTASTAIN Elite Avidin:Biotinylated enzyme Complex (ABC) Kit (Vectorlabs, Burlingame, CA, USA). Air-dried slides with 8-μm sections were fixed in methanol with 3% hydrogen peroxide to quench endogenous peroxidases and then blocked with serum for 20 minutes. Incubation with HOXA4 primary antibody (catalog number ab26097, Abcam Cambridge, MA), secondary antibody (catalog number sc-2054, Santa Cruz Biotechnology, Inc., Santa Cruz, CA, USA), ABC reagent, and diaminobenzidine substrate were performed according to the manufacturer's instructions. Each treatment was preceded by a 10-minute PBS wash. Immunostained slides were counterstained with hematoxylin and mounted with a coverslip. Non-specific IgG antibody in lieu of primary antibody served as a negative control.

When using formalin-fixed paraffin embedded aortic tissue, 5-μm sections were used in a protocol described previously [10]. The slides were incubated with HOXA4 primary antibody [15] (ab26097 from Abcam) on an automatic immunostainer (Autostainer, DAKO, Carpinteria, CA). A secondary antibody with avidin-biotin peroxidase amplification from DAKO was used and the signal detected using diaminobenzidine as a chromogen. Images were obtained using either a Leica DM4000B (Leica Microsystems, Inc., Bannockburn, IL, USA) or Nikon (Nikon Instruments Inc., Melville, NY, USA) microscope.

### Western blot analysis of protein lysates from cultured cells

Human aortic SMCs (SMC1 and SMC2), human aortic endothelial cells (EC1 and EC2), and monocyte/macrophage cells (MP1 and MP2) listed in Additional file 4, Table S3 were cultured in appropriate medium according to suppliers' recommendations. Aortic ECs and SMCs were stimulated for 18 hours using 50 ng/ml IFN-γ (Peprotech) [16]. Monocyte/Macrophage cell lines were stimulated 18 hours using 100 ng/ml LPS (serotype 055:B5, Sigma), and 20 ng/ml IFN-γ [17]. During stimulation, serum concentrations were half the concentrations used for maintenance culture. This stimulation was considered relevant to AAA, since mice lacking IFN-γ are resistant to AAA formation in the CaCl_2 _model [18] and IFN-γ-producing T cells are present in the blood and aortic wall of most AAA patients [19]. Cells were washed twice with cold PBS and lysed in RIPA buffer with included protease inhibitors (Santa Cruz, sc-24948). Cytoplasmic and nuclear extracts were also prepared using NE-PER Nuclear and Cytoplasmic Extraction Reagents (Product No. 78833, Pierce Biotechnology, Rockford, IL, USA) according to the manufacturer's recommendations. Cell lysates were subjected to SDS-PAGE, transferred to PVDF membrane and immunoblotted with anti-HOXA4 rabbit polyclonal antibody (Abcam, ab26097) or anti-ACTB mouse monoclonal antibody (Sigma, A1978).

Blots were quantified to obtain an intensity-per-area value for each band. Intensity values were adjusted for ACTB protein levels. ACTB-adjusted band intensity values were input into the R statistical package [13]. Repeated measures mixed effects linear model (R package lme4), was used to evaluate the effect of inflammatory stimulation on HOXA4 levels.

### Fluorescence microscopy on cultured cells

Human aortic SMCs (SMC1 and SMC2, Additional file 4, Table S3) and human aortic ECs (EC1 and EC2, Additional file 4, Table S3) were grown on 2-well Permanox chamber slides (Lab-Tek, Thermo Fisher Scientific, Rochester, NY, USA), fixed with 3% paraformaldehyde and permeabilized with 0.05% Triton X-100 in PBS, and then incubated with rhodamine-phalloidin (Molecular Probes, Invitrogen Corporation, Carlsbad, CA, USA) at 1:100 dilution, and anti-HOXA4 with 1:200 dilution (ab26097, Abcam), followed by incubation with fluorescein-conjugated affinity-purified donkey anti-rabbit IgG (1:100 dilution in BLOTTO; Jackson ImmunoResearch Laboratories Inc., West Grove, PA, USA). The nuclei were stained with DAPI. The stained cells were examined using a Zeiss Observer Z1 inverted light microscope equipped for epifluorescence.

## Results

### Regional differences in the expression of HOX genes in the baboon aorta

To identify novel candidate genes with spatial expression differences in the aorta, genome-wide expression profiling was undertaken using segments along the entire aorta (Figure 1) from baboons (*Papio hamadryas anubis*), which have been used as a model organism for vascular disease [20]. Aortic tissue from the baboons was preserved immediately following euthanasia and high-quality RNA was subsequently extracted. As no baboon-specific microarrays were available, RNA was hybridized to human arrays. Without a completed baboon genome the full extent of sequence conservation is not known, but over 95% conservation would be expected based on sequence similarity between humans and other primate species. For highly conserved genes such as the HOX family of genes, this degree of conservation is expected; *HOXA4 *coding region sequence identity is 97.4% (938/963 nucleotides) and protein sequence identity is 96.6% (309/320 amino acids) between humans and baboons.

Genes with increasing or decreasing trends in expression along the baboon aorta included several members of the HOX gene family. Figure 1 shows the regional expression of *HOXA4 *in two baboon aortas separately (dashed and solid lines).

### Differences in the expression of HOX genes in human abdominal aortic aneurysms compared to matched control aortas

**Figure 2:**
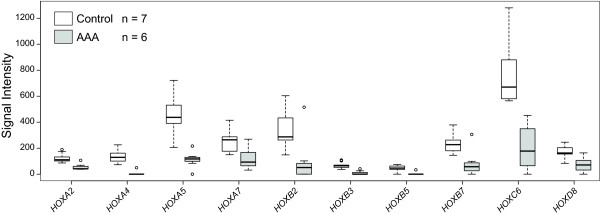
**HOX genes with significant differences between human AAA and control human abdominal aorta assayed by microarrays**. Box-and-whisker plots of mRNA levels for the 10 classic HOX genes that had significantly (FDR <0.05) different expression levels between AAA cases (N = 6) and age-, sex-, and ethnicity-matched controls (N = 7) obtained from abdominal aorta. Results are based on microarray expression profiling which have been described previously [11] and details on the donors are described in Additional file 2, Table S2. RNA expression levels in signal intensity units are indicated on the ordinate-axis. Thick horizontal bars in the boxes indicate median values, boxes indicate interquartile range, whiskers indicate range of non-outlier values, and circles indicate outliers less than 3 interquartile range units. Gene symbols available from the National Center for Biotechnology Information (NCBI; http://www.ncbi.nlm.nih.gov/) were used. For a full list of all HOX genes and their expression levels in human aorta, see Additional file 5, Table S4.

The regional expression of HOX genes in the baboon aorta led us to hypothesize that HOX gene expression might be altered in human AAA. Indeed, using microarray data generated previously [11], we found 78 homeodomain-containing genes which were expressed in the human aorta (Additional file 5, Table S4); 31 of these had significantly different mRNA levels (Additional file 5, Table S4) in AAA compared to control abdominal aortic tissues from an age-, sex-, and ethnicity-matched group of individuals (Additional file 2, Table S2). Ten of the 31 differentially expressed homeodomain-containing genes were classic HOX-genes; the mRNA levels of all of them were significantly decreased in AAA (Figure 2).

**Figure 3:**
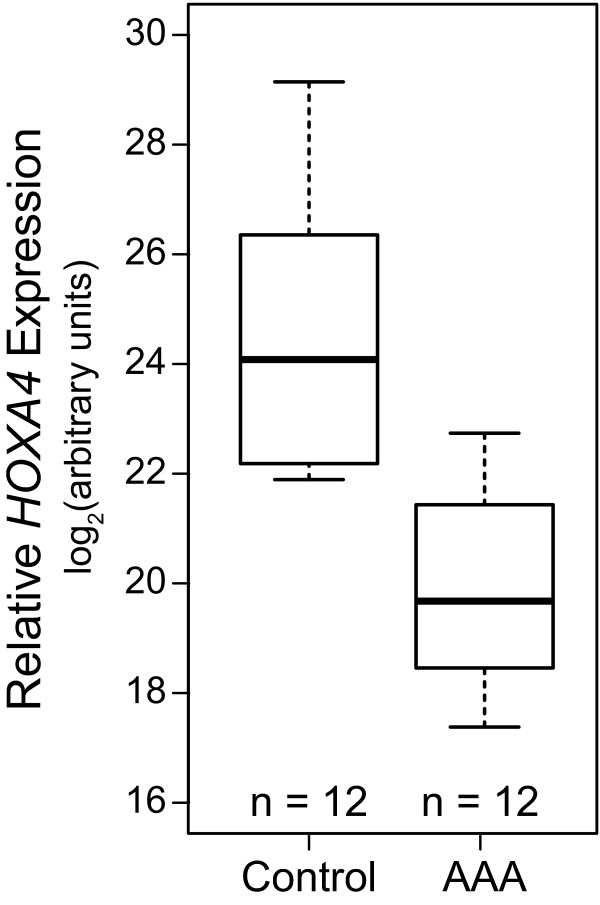
**Decreased levels of *HOXA4 *mRNA in human AAA**. *HOXA4 *mRNA levels in human AAA (N = 12), and control human abdominal aorta (N = 12) were assayed by Q-RT-PCR. For details on the donors, see Additional file 2, Table S2. The difference in *HOXA4 *mRNA levels between AAA and control samples was statistically significant (*P *< 0.00004).

*HOXA4 *was the most significantly decreased HOX gene in human AAA tissue based on microarray results. In a different set of 12 AAA cases and 12 similarly-aged controls (case mean = 69.9 +/- 10.2 y; control mean = 64.3 +/- 14 y; *P *= 0.27; Additional file 2, Table S2) real-time quantitative RT-PCR was used to confirm the decrease in *HOXA4 *mRNA expression (*P *< 0.00004; Figure 3). We also detected *HOXA4 *mRNA in cultured human aortic SMCs and aortic ECs (data not shown).

### Regional differences in the abundance of *HOXA4 *protein in the human aorta

**Figure 4:**
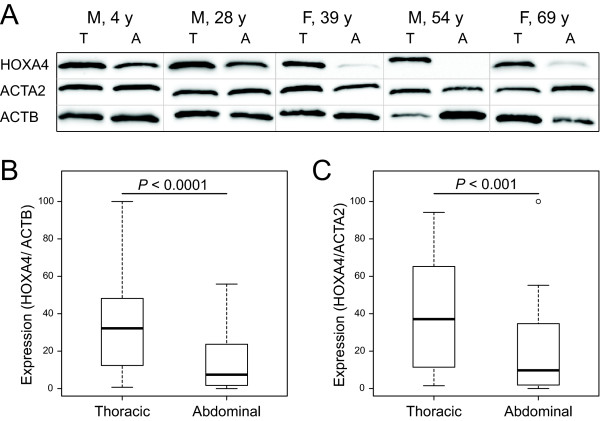
**HOXA4 protein abundance in human aorta**. A, Representative images of western blots with paired thoracic (T) and abdominal (A) aorta of the same person; B and C, Comparison of HOXA4 protein levels between 24 pairs of thoracic and abdominal aortic samples standardized to either β-actin (ACTB; B) or α-actin (ACTA; C). Clinical information is available in Additional file 1, Table S1.

Western blot analysis using paired human thoracic and abdominal aortic samples (n = 48 from 24 individuals with ages ranging from 4 to 78 years, Additional file 1, Table S1) was performed to determine whether the observed spatial differences in baboon mRNA expression were reflected in differences in protein levels in human aorta. In non-aneurysmal aortic tissue HOXA4 was detected using a specific antibody [15] as a single band at ~33 kDa (Figure 4 and Additional file 3, Figure S1). After adjusting for β-actin (ACTB), the level of HOXA4 protein was significantly higher in thoracic than the abdominal aorta (*P *< 0.0001; Figures 4A and 4B), consistent with the baboon microarray analysis (Figure 1). Smooth muscle α-actin (ACTA2) protein levels were also investigated to assess whether the difference in the abundance of HOXA4 was a result of higher SMC density in the thoracic aorta. After adjusting for α-actin levels, HOXA4 remained significantly higher in the thoracic aorta than in the abdominal aorta (*P *< 0.001; Figures 4A and 4C).

**Figure 5:**
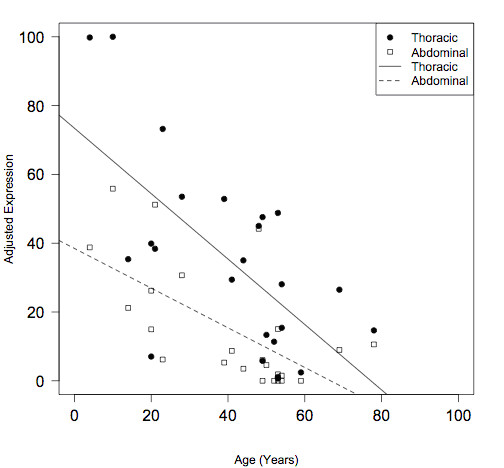
**HOXA4 protein levels decrease with age**. Using ACTB-adjusted HOXA4 protein levels, regression lines were calculated for age versus thoracic or abdominal aortic protein levels. Slopes were significantly different from zero. Thoracic: slope = -0.95 (99% C.I. -1.66, -0.24); R^2^ = 0.43; F-statistic *P *= 0.0005. Abdominal: slope = -0.58 (99% C.I. -1.02, -0.13); R^2^ = 0.41; F-statistic *P *= 0.00075.

The potential effects of age, race and sex (factors known to modify AAA risk) on HOXA4 protein levels were also investigated. Linear regression with stepwise backwards elimination showed age to be the only significant predictor of HOXA4 protein abundance, with evidence that protein levels decrease with age (negative correlation; Figure 5).

### Immunohistochemical localization of HOXA4 in aortic tissue samples

**Figure 6:**
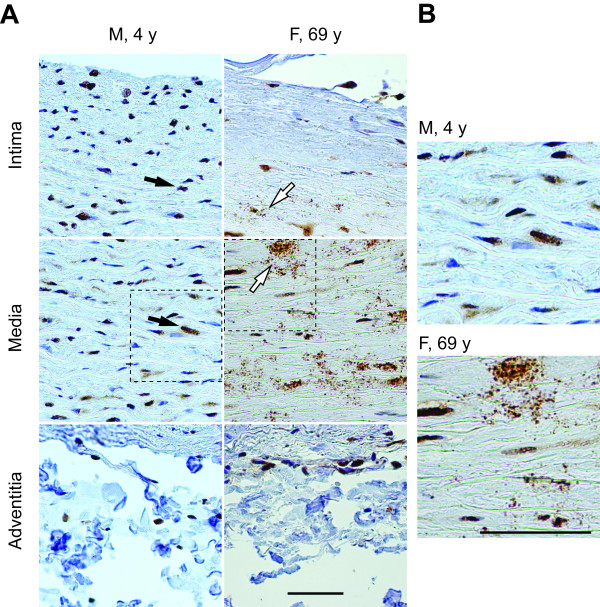
**Immunohistochemical staining for HOXA4 in human aorta**. A, Representative images of intima, media, and adventitia of young and aged aortic tissue. Arrows indicate nuclear staining (black arrows) and punctuate, aggregate-like staining (white arrows). B, Enlarged frames of the boxed regions in A. Scale bar = 50 μm.

Formalin-fixed paraffin-embedded samples of human aortic tissues were used for immunohistochemical staining. HOXA4 staining was observed in the nuclei and perinuclear regions of several cell types in all three layers of the aorta (Figure 6). Staining patterns appeared to change with age. In samples from both young and old individuals staining was observed in and around the nuclei, but staining tended to concentrate more in the nuclei of samples from young individuals whereas samples from older individuals showed more diffuse perinuclear staining (Figure 6). Additionally, an intense punctate staining pattern was observed in samples from older individuals that was not present in younger samples (Figure 6).

Similar staining patterns, including the punctate staining, were also observed with frozen tissue sections (i.e. non-fixed tissue; not shown), demonstrating that it was not an artifact related to tissue preparation.

### Characterization of HOXA4 expression in cultured vascular cells

**Figure 7:**
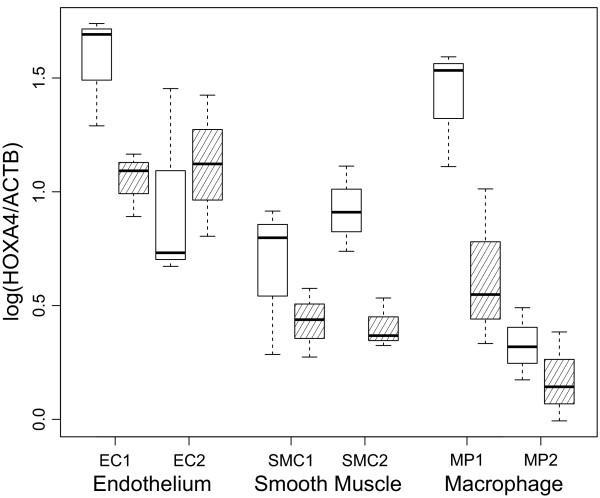
**Inflammatory stimulation leads to decrease in HOXA4 protein in cultured cells**. Human aortic endothelial cells (EC1 and EC2), and SMCs (SMC1 and SMC2) were stimulated with INF-γ, and macrophages (MP1 and MP2) were stimulated with LPS and INF-γ, and assayed for HOXA4 protein by Westerns blots. Results were standardized with β-actin protein (ACTB) levels. Shaded boxes represent stimulated cells. All but one of the cell lines (EC2) exhibited downregulation of HOXA4 levels within 18 hours of exposure to inflammatory stimuli. The decrease in HOXA4 levels was statistically significant (*P *< 0.0007).

Since inflammation is a hallmark of AAA pathogenesis [2], we examined whether inflammatory stimuli altered levels of HOXA4 in cultured human vascular cells. Human macrophage/monocytes (MP1 and MP2) were stimulated using both IFN-γ and LPS, whereas human aortic ECs (EC1 and EC2), and human aortic SMCs (SMC1 and SMC2) were stimulated with IFN-γ. HOXA4 protein levels were analyzed by western blots and compared with unstimulated control cells. This stimulation was considered relevant to AAA, since mice lacking IFN-γ are resistant to AAA formation in the CaCl_2 _model [18] and IFN-γ-producing T cells are present in the blood and aortic wall of most AAA patients [19]. All but one of the cell lines (EC2) exhibited downregulation of HOXA4 levels within 18 hours of exposure to inflammatory stimuli (Figure 7). The decrease in HOXA4 levels was statistically significant (*P *< 0.0007).

When investigating the cytoplasmic and nuclear fractions of proteins separately, the relative amount of HOXA4 protein (adjusted to ACTB protein levels) in the nucleus decreased with inflammatory stimulation (*P *= 0.0048; paired t test) in all cell types.

**Figure 8:**
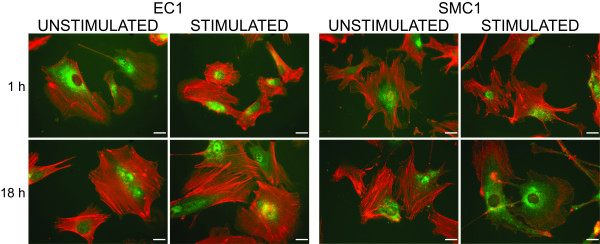
**Localization of HOXA4 protein in cultured human aortic ECs (A) and SMCs (B) using immunofluorescence**. The cells were stimulated for 1 or 18 hours using 50 ng/ml IFN-γ. Green, staining with anti-HOXA4 antibody; and Red, F-actin filaments stained with phalloidin. Scale bar = 20 μm. See Additional file 6, Figure S2 for images with DAPI stain.

Immunofluorescent staining for HOXA4 was also performed using cultured human aortic EC and human aortic SMCs. In both cell types the strongest signal was observed in the perinuclear region with a small amount of nuclear staining (Figure 8, and Additional file 6, Figure S2). Consistent with previously published results, stimulated SMCs had fewer large actin stress fibers visualized by co-staining with rhodamine-phalloidin, whereas stress fibers in ECs did not decrease with stimulation [21,22].

## Discussion

The results of the current study provide evidence of differences in gene expression between the descending thoracic and abdominal aorta, particularly among the HOX family of transcription factors. Additionally, several HOX transcription factors demonstrated altered expression in human AAA compared to a group of age-, race- and sex-matched control aorta, with *HOXA4 *being the most downregulated. Quantitative RT-PCR using a separate set of samples confirmed that *HOXA4 *mRNA levels were decreased in AAA and western blots demonstrated that protein levels were lower in the human abdominal than thoracic aorta. Immunohistochemical staining suggested expression of HOXA4 in SMCs and ECs in aortic tissue sections. Finally, HOXA4 was observed in the nuclear and perinuclear regions of cultured aortic ECs and SMCs with decreased protein levels after stimulation with inflammatory cytokines.

HOX genes were originally identified for their functions in embryonic patterning. Increasing evidence suggests, however, that these genes also have a role in maintaining spatial identity of tissues, including vasculature, as demonstrated by microarray analyses [6,7] and animal models [8]. The results of our microarray analyses using the baboon aortas were consistent with these studies, demonstrating that the expression of HOX genes varies spatially, in particular between the more aneurysm-susceptible abdominal aorta and the less aneurysm-susceptible thoracic aorta. Several of these HOX genes have been implicated in vascular function and disease, supporting the hypothesis that regional gene expression could alter disease susceptibility [9,23]. A recent study supports this hypothesis, with *HOXA1 *exhibiting higher expression at atherosusceptible sites in the porcine aorta [24].

To assess the potential role of HOX genes in AAA pathogenesis, their expression profiles from human AAA and control aorta were compared, demonstrating significant downregulation of 10 classic HOX genes in AAA tissue. These findings are consistent with previous array-based studies, which demonstrated downregulation of HOX genes in atherosclerosis and ulcerative colitis compared with non-diseased aorta and colon, respectively [25,26]. Protein analyses described here using cultured human vascular cells demonstrated a decrease in HOXA4 protein levels after *in vitro *stimulation with IFN-γ or LPS and show that inflammatory stimuli alter HOX gene expression. Previously, treatment with interleukin 1ß (IL1B), an inflammatory cytokine implicated in AAA pathogenesis [2], was shown to downregulate several HOX genes [27]. Downregulation of HOX genes could have several detrimental effects. HOX genes have well-established roles in regulating cell proliferation and differentiation [9,23]; downregulation of HOX genes could alter cell phenotype in AAA. Decreased HOX gene expression could alter transcriptional regulation; HOX gene targets include *SPP1 *[28,29] and the matrix metallopeptidases [30], which have been implicated in AAA pathogenesis [2].

In the current study we concentrated on *HOXA4*, since it exhibited the most significant downregulation in human AAA tissue, had a distinct regional expression pattern along the aorta, and a specific antibody against it was available. Western blots confirmed regional variation in HOXA4 protein abundance, with significantly higher HOXA4 levels in human thoracic aorta compared with abdominal aorta. Immunohistochemical staining suggested that multiple cell types in the aorta express HOXA4, primarily ECs, and SMCs, as well as some adventitial fibroblasts. Immunofluorescent staining of cultured ECs and SMCs showed nuclear and perinuclear protein localization of HOXA4. *HOXA4 *expression in both ECs and SMCs is interesting, as human *HOXA9 *and murine *Hoxa3 *are also expressed in both endothelium and smooth muscle, whereas murine *Hoxc11 *is expressed in the SMC only, suggesting that expression in both EC and SMC is characteristic of the HOXA gene cluster [8].

Similar to other *HOX *genes, *HOXA4 *is involved in embryonic patterning. Its function in embryogenesis does not appear to be critical, since targeted disruption of *Hoxa4 *results in a relatively mild phenotype; *Hoxa4 *knockout mice are viable and exhibit only some deformations of the cervical spine [31,32]. Conversely, transgenic mice overexpressing the *Hoxa4 *gene develop congenital megacolon due to abnormalities in the enteric nervous system [33]. Studies in chick embryos suggest that *Dfd*, the *HOXA4*-like gene in chickens, is not critical to aortic arch artery patterning, since treatment of the cardiac neural fold with a *Dfd *antisense oligonucleotide resulted in no apparent change in heart development at stage 18 to 24 [34].

It is possible that *HOXA4 *is involved in regulating angiogenic function similar to other HOX genes. An *in vitro *comparison of ovarian carcinoma cell lines showed that cells exhibiting higher levels of *HOXA4 *expression have decreased motility by scratch assay [15]. Similarly, overexpression of *HOXA4 *further decreased cell motility and led to a corresponding increase in ß1 integrin [35]. ß1 integrin has been implicated in AAA pathogenesis and is decreased in AAA tissues compared to non-aneurysmal aorta [36]. siRNA knockdown of *HOXA4 *does not ablate ß1 integrin expression, however, which is consistent with HOX genes having redundant functions [35]. A role for HOXA4 in mediating cell contacts is further supported by an increase in basal lamina thickness and collagen in the colonic mucosa of transgenic mice overexpressing *Hoxa4 *[33]. It is, therefore, plausible that loss of HOXA4 function in medial SMCs contributes to adverse phenotypic changes in aneurysms, including disruption of cell-cell interactions. HOXA4 can bind to its own promoter region and there is some evidence for *HOXA4 *regulating the adjacent *HOXA5 *gene under certain conditions [37,38]. Additionally, *HOXA4 *has also been implicated in the regulation of microRNA genes during monocyte differentiation, suggesting microRNAs as one mechanism for HOX gene regulation of cell-type specific effects [39].

## Conclusions

Our results suggest that *HOXA4 *is important for maintaining spatial identity even in the adult aorta and that downregulation of *HOXA4 *expression may increase susceptibility to aortic disease such as AAAs. To test these hypotheses embryonic targeted deletions of HOXA4 in animal models are needed to determine the exact contribution of this gene family to maintaining spatial identity. Similarly, targeted deletion mutants could be employed to prove that downregulation of HOXA4 increases susceptibility to AAA in a mouse model.

## Authors' contributions

JHL participated in the design of the study, analyzed microarray data, carried out western blot experiments with human tissue lysates, and drafted the manuscript. RE carried out all experiments with cultured cells and prepared illustrations. CMS and ZG carried out and interpreted the immunohistochemical analyses. AG and KD catalogued phenotypic information, processed samples, and carried out RT-PCRs. LAC provided baboon samples. JS and LL interpreted results. RSVH and LH provided control samples and interpreted results. GML participated in the design of the study, analyzed microarray data and interpreted results. JRE, DPF, JLG and RPG recruited AAA patients for the study and collected tissue specimens. DJC and WDL interpreted results. GT participated in the design of the study, analyzed results, carried out statistical analyses, prepared illustrations and interpreted results. HK obtained funding for the study, participated in the design of the study, interpreted results, and participated in drafting the manuscript. All authors read and approved the final manuscript.

## Supplementary Material

Additional file 1 **Table S1. Human tissue samples from non-aneurysmal aortas used in assessing protein abundance of HOXA4**. List of sample IDs with donor age, ethnicity, sex and cause of death for human samples used to study HOXA4 protein levels. Click here for file

Additional file 2 **Table S2. Human aortic tissue samples used in microarray and Q-RT-PCR experiments**. List of sample IDs with donor disease status classification, ethnicity, sex, age, and cause of death for human samples used in microarray and RT-PCR studies. Click here for file

Additional file 3 **Figure S1. Specificity of HOXA4, ACTA2 and ACTB antibodies**. Images of western blots performed with antibodies used in the study. Click here for file

Additional file 4 **Table S3. Commercially available human cell lines for HOXA4 experiments**. List of cell lines used in the study with codes, description of cell type, vendor, and catalog number. Click here for file

Additional file 5 **Table S4. mRNA expression of HOX genes in human aneurysmal (N = 6) and non-aneurysmal (N = 7) abdominal aorta based on microarray analyses**. List of differentially expressed homeodomain-containing genes in human AAA compared to age-sex-and ethnicity-matched controls. Columns give the full protein name, Gene Symbol, GeneID, expression levels in control group and AAA group, fold-change in expression, False Discovery Rate and Illumina Detection Score in control and AAA group. Click here for file

Additional file 6 **Figure S2. Localization of HOXA4 protein in cultured human aortic ECs (A) and SMCs (B) using immunofluorescence**. Images of stained cultured cells. Click here for file
