# Supplementary material for: Regional expression of HOXA4 along the aorta and its potential role in human abdominal aortic aneurysms
Source: BMC Physiol. 2011 May 31;11:9. doi: 10.1186/1472-6793-11-9 (PMC3125234; doi:10.1186/1472-6793-11-9)
Supplement: Additional file 1 — Table S1. Human tissue samples from non-aneurysmal aortas used in assessing protein abundance of HOXA4. List of sample IDs with donor age, ethnicity, sex and cause of death for human samples used to study HOXA4 protein levels. [file 1472-6793-11-9-S1.PDF]

Additional file 1, Table S1. Human tissue samples from non-aneurysmal aortas used in assessing protein abundance of HOXA4

Case ID	Age (years)	Ethnicity*	Sex	Cause of Death†
1	21	C	M	Overdose
2	50	AA	M	Overdose
3	78	C	M	CA
4	4	AA	M	Unknown
5	41	AA	M	Heart Failure
6	28	AA	M	CA
7	54	C	M	CA
8	69	C	F	Trauma
9	52	C	F	CA
10	59	C	F	CA
11	54	AA	M	GSW
12	39	C	F	Unknown
13	53	C	M	GSW
14	14	AA	F	Suicide
15	53	C	M	Overdose
16	20	AA	M	GSW
17	10	AA	M	GSW
18	48	AA	F	Trauma
19	20	AA	M	Trauma
20	23	AA	M	GSW
21	49	AA	M	CA
22	49	AA	F	Overdose
23	53	AA	M	CA
24	44	C	M	Overdose

*C. Caucasian; AA, African American

†GSW, gunshot wound; CA, cardiac arrest
