# Supplementary material for: Regional expression of HOXA4 along the aorta and its potential role in human abdominal aortic aneurysms
Source: BMC Physiol. 2011 May 31;11:9. doi: 10.1186/1472-6793-11-9 (PMC3125234; doi:10.1186/1472-6793-11-9)
Supplement: Additional file 2 — Table S2. Human aortic tissue samples used in microarray and Q-RT-PCR experiments. List of sample IDs with donor disease status classification, ethnicity, sex, age, and cause of death for human samples used in microarray and RT-PCR studies. [file 1472-6793-11-9-S2.PDF]

Additional file 2.
Table S2. Human aortic tissue samples used in microarray and Q-RT-PCR experiments

Case ID	Tissue Type	Ethnicity	Sex	Age (years)	Cause of Death	Microarray	Q-RT-PCR
A1-F	AAA	C	F	82		√	
A2-F	AAA	C	F	68		√	
A3-F	AAA	C	F	64		√	
A2-M	AAA	C	M	63		√	
A3-M	AAA	C	M	67		√	
A4-M	AAA	C	M	63		√	
C1-F	Control	C	F	74	Cancer	√	
C2-F	Control	C	F	52	Cancer	√	
C3-F	Control	C	F	84	Aortic arch dissection	√	
C1-M	Control	C	M	65	Peritonitis	√	
C2-M	Control	C	M	59	Cancer	√	
C3-M	Control	C	M	52	Liver cirrhosis	√	
C4-M	Control	C	M	73	Cancer	√	
GHS000001	AAA	C	M	67			√
GHS000005	AAA	C	M	64			√
GHS000002	AAA	C	F	63			√
GHS000003	AAA	C	M	88			√
GHS000006	AAA	C	F	81			√
GHS000007	AAA	C	F	65			√
GHS000011	AAA	C	F	61			√
GHS000012	AAA	C	M	70			√
GHS200106	AAA	C	M	87			√
GHS200210	AAA	C	M	60			√
GHS200212	AAA	C	M	73			√
GHS200214	AAA	C	M	60			√
NDRI56735	Control	C	F	54	Cancer		√
NDRI56724	Control	C	F	77	Natural		√
NDRI57017	Control	C	F	78	Cardiovascular		√
NDRI57054	Control	C	M	69	Cardiovascular		√
NDRI57179	Control	C	F	57	Respiratory Failure		√
ME0204	Control	AA	M	50	Overdose		√
ME0205	Control	C	M	78	Cardiovascular		√
ME0501	Control	C	F	69	Trauma		√
ME0503	Control	C	M	54	Cardiovascular		√
ME1001	Control	C	F	88	Trauma		√
ME1002	Control	AA	M	53	Overdose/Cardiovascular		√
ME1003	Control	C	M	44	Overdose		√

AAA, abdominal aortic aneurysm; C, Caucasian; AA, African American; F, female; M, male

For the microarray study the summary statistics were:
AAA group (N = 6): mean age = 67.8 +/- 7.3, Median = 65.5, Male:Female = 3:3
Control group (N = 7): mean age = 65.6 +/- 12.1, Median = 65, Male:Female = 4:3
Comparison of ages in the study groups: p = 0.69 (two-tailed t-test, unequal variance)

For the Q-RT-PCRs the summary statistics were:
AAA group (N = 12): mean age = 69.9 +/-10.2; median = 66; Male:Female = 8:4
Control group (N = 12): mean age = 64.3 +/-14; median = 63; Male:Female = 6:6
Comparison of ages in the study groups: p = 0.27 (two-tailed t-test, unequal variance)
