# Supplementary material for: Regional expression of HOXA4 along the aorta and its potential role in human abdominal aortic aneurysms
Source: BMC Physiol. 2011 May 31;11:9. doi: 10.1186/1472-6793-11-9 (PMC3125234; doi:10.1186/1472-6793-11-9)
Supplement: Additional file 3 — Figure S1. Specificity of HOXA4, ACTA2 and ACTB antibodies. Images of western blots performed with antibodies used in the study. [file 1472-6793-11-9-S3.PDF]

Additional file 3.

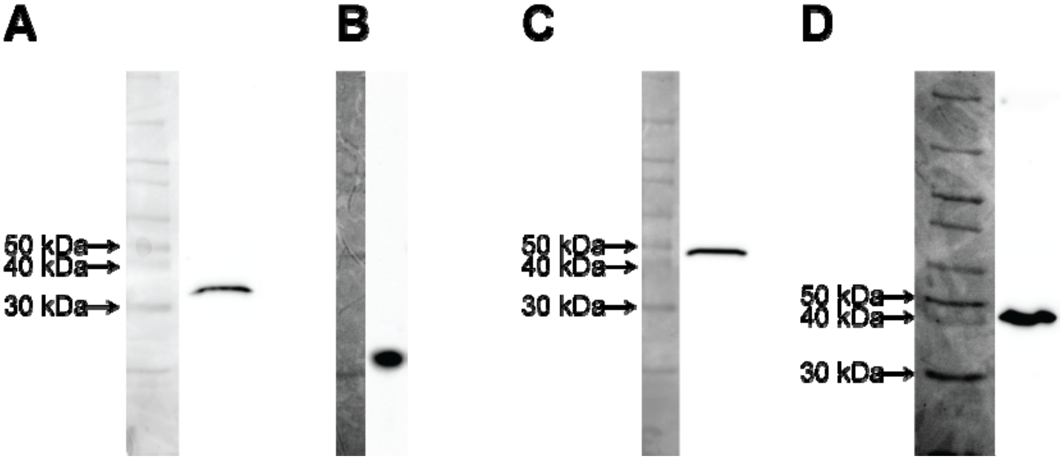

Figure S1. Specificity of HOXA4, ACTA2 and ACTB antibodies. Protein lysates from human aortic wall tissue samples (A, C, D) and HOXA4 immunizing peptide (B) were run on PAGE, blotted onto membranes and used for Western analysis. Bands for HOXA4 (A), ACTA2 (C), and ACTB (D) were detected at the expected molecular weights of ~33 kDa, ~42 kDa, and ~42 kDa, respectively. The HOXA4 immunizing peptide (ab32311, Abcam Inc., Cambridge, MA) gave a band of the expected size with the HOXA4 antibody (B). The HOXA4 antibody has also been used by others (Ota et al. 2009). Left-side lane in each figure is molecular weight marker (Novex) with sizes indicated on the left, and the right-side lane is the immunostained protein.

Ota T, Klausen C, Salamanca MC, Woo HL, Leung PC, Auersperg N. Expression and function of HOXA genes in normal and neoplastic ovarian epithelial cells. Differentiation. 2009;77:162-171.
