# Supplementary material for: Regional expression of HOXA4 along the aorta and its potential role in human abdominal aortic aneurysms
Source: BMC Physiol. 2011 May 31;11:9. doi: 10.1186/1472-6793-11-9 (PMC3125234; doi:10.1186/1472-6793-11-9)
Supplement: Additional file 4 — Table S3. Commercially available human cell lines for HOXA4 experiments. List of cell lines used in the study with codes, description of cell type, vendor, and catalog number. [file 1472-6793-11-9-S4.PDF]

Additional file 4, Table S3. Commercially available human cell lines for HOXA4 experiments

Code	Cell Type	Vendor	Cat #
SMC1	Aortic smooth muscle	ScienCell	6110
SMC2	Aortic smooth muscle	ATCC	CRL-1999
EC1	Aortic endothelial	ScienCell	6100
EC2	Aortic endothelial	PromoCell	C-12271
MP1	Monocyte/Macrophage	ATCC	TIB-202
MP2	Monocyte/Macrophage	ATCC	CRL-9850
