# Supplementary material for: Regional expression of HOXA4 along the aorta and its potential role in human abdominal aortic aneurysms
Source: BMC Physiol. 2011 May 31;11:9. doi: 10.1186/1472-6793-11-9 (PMC3125234; doi:10.1186/1472-6793-11-9)
Supplement: Additional file 5 — Table S4. mRNA expression of HOX genes in human aneurysmal (N = 6) and non-aneurysmal (N = 7) abdominal aorta based on microarray analyses. List of differentially expressed homeodomain-containing genes in human AAA compared to age-sex-and ethnicity-matched controls. Columns give the full protein name, Gene Symbol, GeneID, expression levels in control group and AAA group, fold-change in expression, False Discovery Rate and Illumina Detection Score in control and AAA group. [file 1472-6793-11-9-S5.PDF]

Additional file 5, Table S4. mRNA expression of HOX genes in human aneurysmal (N=6) and non-aneurysmal (N=7) abdominal aorta based on microarray analyses*

			Expression*				Detection Score†	
HOX Protein	Symbol	GeneID	Control	AAA	FC‡	FDR§	Control	AAA
activity-dependent neuroprotector	ADNP	23394	13.49	30.26	2.24	0.079	0.94	0.99
AT-binding transcription factor 1	ATBF1	463	337.98	130.53	-2.59	0.0106	1	1
bagpipe homeobox homolog 1 (Drosophila)	BAPX1	579	0.56	26.29	46.95	0.10	0.48	0.99
BarH-like 2 (Drosophila)	BARHL2	343472	29.84	19.01	-1.57	0.26	0.99	0.98
caudal type homeobox transcription factor 4	CDX4	1046	23.87	36.21	1.52	0.34	0.98	0.99
cut-like 1, CCAAT displacement protein (Drosophila)	CUTL1	1523	74.2	57.4	-1.29	0.47	0.99	0.99
distal-less homeobox 1	DLX1	1745	27.37	26.44	-1.04	0.70	0.99	0.99
double homeobox, 3	DUX3	26582	60.8	50.99	-1.19	0.46	0.99	0.99
double homeobox 4c	DUX4C	554045	3.34	23.53	7.04	0.0220	0.67	0.99
eve, even-skipped homeobox homolog 1 (Drosophila)	EVX1	2128	33.96	41.38	1.22	0.50	0.99	0.99
homeobox, ES cell expressed 1	HESX1	8820	13.33	24.7	1.85	0.27	0.94	0.99
homeobox, hematopoietically expressed	HHEX	3087	168.5	403.07	2.39	0.11	1	1
homeodomain interacting protein kinase 2	HIPK2	28996	58.73	58.22	-1.01	0.72	0.99	0.99
H2.0-like homeobox 1 (Drosophila)	HLX1	3142	525.21	692.31	1.32	0.22	1	1
homeodomain-only protein	HOP	84525	92.54	107.77	1.16	0.60	0.99	1
homeobox A2	HOXA2	3199	111.28	61.95	-1.80	0.0106	1	0.99
homeobox A4	HOXA4	3201	132.46	10.23	-12.95	1.08E-07	1	0.92
homeobox A5	HOXA5	3202	423.48	122.85	-3.45	1.34E-05	1	1
homeobox A6	HOXA6	3203	85.04	73.25	-1.16	0.50	0.99	0.99
homeobox A7	HOXA7	3204	252.03	132.49	-1.90	0.034	1	1
homeobox A9	HOXA9	3205	79.17	16.28	-4.86	0.16	0.99	0.97
homeobox A10	HOXA10	3206	41.75	25.15	-1.66	0.17	0.99	0.99
homeobox A11	HOXA11	3207	18.17	25.38	1.40	0.38	0.97	0.99
homeobox B2	HOXB2	3212	298.94	108.01	-2.77	0.030	1	1
homeobox B3	HOXB3	3213	64.09	22.43	-2.86	0.00031	0.99	0.99
homeobox B4	HOXB4	3214	54.87	19.56	-2.81	0.064	0.99	0.98
homeobox B5	HOXB5	3215	42.06	6.64	-6.33	0.00023	0.99	0.84
homeobox B7	HOXB7	3217	217.93	86.42	-2.52	0.0098	1	0.99
homeobox C5	HOXC5	3222	36.3	10.93	-3.32	0.17	0.99	0.94
homeobox C6	HOXC6	3223	764.35	219	-3.49	9.13E-05	1	1
homeobox C10	HOXC10	3226	146.46	89.22	-1.64	0.39	1	1
homeobox D8	HOXD8	3234	167.1	78.34	-2.13	0.0109	1	0.99
iroquois homeobox protein 1	IRX1	79192	68.9	5.06	-13.62	0.0119	0.99	0.75
iroquois homeobox protein 3	IRX3	79191	80.31	65.03	-1.23	0.55	0.99	0.99
iroquois homeobox protein 6	IRX6	79190	29.24	48.35	1.65	0.14	0.99	0.99
iroquois homeobox protein-like 1	IRXL1	283078	407.27	77.52	-5.25	2.63E-07	1	0.99
LIM homeobox 3	LHX3	8022	71.16	46.58	-1.53	0.095	0.99	0.99
LIM homeobox 4	LHX4	89884	172.34	198.59	1.15	0.55	1	1
LIM homeobox 6	LHX6	26468	67.17	62.14	-1.08	0.68	0.99	0.99
Meis1, myeloid ecotropic viral integration site 1 homolog (mouse)	MEIS1	4211	293.54	95.54	-3.07	1.45E-05	1	1
Meis1, myeloid ecotropic viral integration site 1 homolog 2 (mouse)	MEIS2	4212	367.16	121.78	-3.01	1.54E-05	1	1
mesenchyme homeobox 1	MEOX1	4222	80.97	58.14	-1.39	0.48	0.99	0.99
mesenchyme homeobox 2	MEOX2	4223	343.81	107.04	-3.21	0.0199	1	1
msh homeobox homolog 1 (Drosophila)	MSX1	4487	347.91	184.62	-1.88	0.0116	1	1
msh homeobox homolog 2 (Drosophila)	MSX2	4488	10.33	21.52	2.08	0.30	0.89	0.99
NK2 transcription factor related, locus 3 (Drosophila)	NKX2-3	159296	12.82	76.82	5.99	0.13	0.93	0.99
NK3 transcription factor related, locus 1 (Drosophila)	NKX3-1	4824	25.93	66.54	2.57	0.038	0.99	0.99
likely ortholog of mouse NOBOX	NOBOX	135935	38.53	33.44	-1.15	0.54	0.99	0.99
pre-B-cell leukemia transcription factor 1	PBX1	5087	123.47	20.53	-6.01	2.19E-09	1	0.99
pre-B-cell leukemia transcription factor 2	PBX2	5089	250.15	341.02	1.36	0.36	1	1
pre-B-cell leukemia transcription factor 3	PBX3	5090	206.25	280.17	1.36	0.17	1	1
pre-B-cell leukemia transcription factor 4	PBX4	80714	4.54	25.36	5.59	0.057	0.71	0.99
pre-B-cell leukemia transcription factor interacting protein 1	PBXIP1	57326	369.88	236.88	-1.56	0.092	1	1
paired-like homeodomain transcription factor 1	PITX1	5307	17.17	21.19	1.23	0.53	0.97	0.99
PBX/knotted 1 homeobox 1	PKNOX1	5316	52.71	56.5	1.07	0.64	0.99	0.99
PBX/knotted 1 homeobox 2	PKNOX2	63876	32.53	15.77	-2.06	0.14	0.99	0.97
POU domain, class 2, transcription factor 1	POU2F1	5451	335.03	305.39	-1.10	0.55	1	1
POU domain, class 2, transcription factor 2	POU2F2	5452	31.99	83.22	2.60	0.0056	0.99	0.99
POU domain, class 6, transcription factor 1	POU6F1	5463	164.3	65.99	-2.49	0.0124	1	0.99
prospero-related homeobox 1	PROX1	5629	0.93	40.05	43.06	0.20	0.53	0.99
paired related homeobox 1	PRRX1	5396	252.53	92.46	-2.73	0.0191	1	1
paired related homeobox 2	PRRX2	51450	950.63	232.94	-4.08	6.90E-05	1	1
special AT-rich sequence binding protein 1 (binds to nuclear	SATB1	6304	301.37	553.27	1.84	0.0103	1	1

matrix/scaffold-associating DNA's)								
SATB family member 2	SATB2	23314	52.34	20.94	-2.50	0.052	0.99	0.99
serologically defined colon cancer antigen 33	SDCCAG33	10194	197.97	159.89	-1.24	0.31	1	1
short stature homeobox	SHOX	6473	12.52	23.41	1.87	0.23	0.93	0.99
short stature homeobox 2	SHOX2	6474	23.23	27.96	1.20	0.59	0.98	0.99
sine oculis homeobox homolog 5 (Drosophila)	SIX5	147912	352.91	158.27	-2.23	0.0193	1	1
transcription factor 8 (represses interleukin 2 expression)	TCF8	6935	29.36	-0.96	30.58	0.10	0.99	0.43
TGFB-induced factor (TALE family homeobox)	TGIF	7050	445.03	565.4	1.27	0.25	1	1
TGFB-induced factor 2 (TALE family homeobox)	TGIF2	60436	132.67	244.3	1.84	0.0136	1	1
VENT homeobox homolog (Xenopus laevis)	VENTX	27287	27.96	218.32	7.81	0.0021	0.99	1
zinc finger homeobox 1b	ZFHX1B	9839	543.3	691.61	1.27	0.24	1	1
zinc finger homeodomain 4	ZFHX4	79776	30.33	8.52	-3.56	0.094	0.99	0.88
zinc fingers and homeoboxes 1	ZHX1	11244	550.96	344.31	-1.60	0.0145	1	1
zinc fingers and homeoboxes 3	ZHX3	23051	176.55	60.38	-2.92	1.79E-05	1	0.99
zinc finger protein 218	ZNF218	128553	98.89	44.53	-2.22	0.029	1	0.99
zinc finger protein 537	ZNF537	57616	79.95	98.98	1.24	0.46	0.99	1

[*] Data based on Lenk et al. (2007) analysis. Expression values from Illumina Beadstudio software.

[†] llumina Beadstudio software *Detection Score* ≥ 0.99 in either AAA or control group was used as the criteria for a gene to be considered expressed.

[‡] Fold Change, defined as the ratio of AAA to control expression. The standard transformation of converting ratios less than 1 to a negative FC has been applied

[§] FDR= False Discovery Rate. *P*-value for AAA compared to control after correction for multiple testing using the two-step Benjamin and Hochberg correction.

Lenk GM, Tromp G, Weinsheimer S, Gatalica Z, Berguer R, Kuivaniemi H. Whole genome expression profiling reveals a significant role for immune function in human abdominal aortic aneurysms. *BMC Genomics.* 2007;8:237.
