# Supplementary material for: Regional expression of HOXA4 along the aorta and its potential role in human abdominal aortic aneurysms
Source: BMC Physiol. 2011 May 31;11:9. doi: 10.1186/1472-6793-11-9 (PMC3125234; doi:10.1186/1472-6793-11-9)
Supplement: Additional file 6 — Figure S2. Localization of HOXA4 protein in cultured human aortic ECs (A) and SMCs (B) using immunofluorescence. Images of stained cultured cells. [file 1472-6793-11-9-S6.PDF]

Additional file 6.

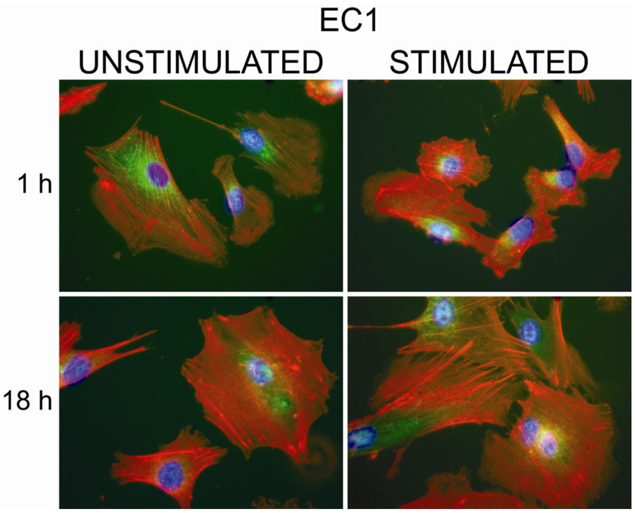

Figure S2A

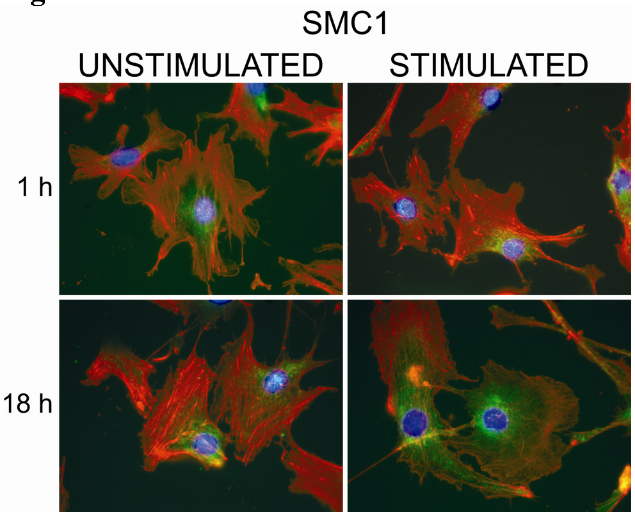

Figure S2B

Figure S2.Localization of HOXA4 protein in cultured human aortic ECs (A) and SMCs (B) using immunofluorescence. The cells were stimulated for 1 or 18 hours using 50 ng/ml IFN-γ. Green, staining with anti-HOXA4 antibody; Red, F-actin filaments stained with phalloidin; and Blue, DAPI stain. For images without DAPI stain, see Figure 8 in the main manuscript.
